# Digital Physical Activity and Exercise Interventions for People Living with Chronic Kidney Disease: A Systematic Review of Health Outcomes and Feasibility

**DOI:** 10.1007/s10916-024-02081-z

**Published:** 2024-07-01

**Authors:** Meg E. Letton, Thái Bình Trần, Shanae Flower, Michael A. Wewege, Amanda Ying Wang, Carolina X Sandler, Shaundeep Sen, Ria Arnold

**Affiliations:** 1 School of Medical, Indigenous & Health Sciences, University of Wollongong, Wollongong, NSW Australia; 2 Neuroscience Research Australia, Randwick, NSW Australia; 3 School of Health Sciences, University of New South Wales, Sydney, NSW Australia; 4 Department of Renal Medicine, Concord Repatriation and General Hospital, Sydney, NSW Australia; 5 Concord Clinical School, University of Sydney, Sydney, NSW Australia; 6 Centre for Pain IMPACT, Neuroscience Research Australia, Sydney, NSW Australia; 7 Renal and Metabolic Division, The George Institute for Global Health, University of New South Wales, Sydney, NSW Australia; 8 The Faculty of Medicine and Health Sciences, Macquarie University, Sydney, NSW Australia; 9 School of Health Sciences, Western Sydney University, Sydney, NSW Australia; 10 The Kirby Institute, University of New South Wales, Sydney, NSW Australia; 11 Menzies Health Institute Queensland, Griffith University, Queensland, Australia

**Keywords:** Chronic kidney disease, Digital health, Physical activity, Exercise, Systematic review

## Abstract

Physical activity is essential to interrupt the cycle of deconditioning associated with chronic kidney disease (CKD). However, access to targeted physical activity interventions remain under-supported due to limited funding and specialised staff. Digital interventions may address some of these factors. This systematic review sought to examine the evidence base of digital interventions focused on promoting physical activity or exercise and their effect on health outcomes for people living with CKD. Electronic databases (PubMed, CINAHL, Embase, Cochrane) were searched from 1 January 2000 to 1 December 2023. Interventions (smartphone applications, activity trackers, websites) for adults with CKD (any stage, including transplant) which promoted physical activity or exercise were included. Study quality was assessed, and a narrative synthesis was conducted. Of the 4057 records identified, eight studies (five randomised controlled trials, three single-arm studies) were included, comprising 550 participants. Duration ranged from 12-weeks to 1-year. The findings indicated acceptability and feasibility were high, with small cohort numbers and high risk of bias. There were inconsistent measures of physical activity levels, self-efficacy, body composition, physical function, and psychological outcomes which resulted in no apparent effects of digital interventions on these domains. Data were insufficient for meta-analysis. The evidence for digital interventions to promote physical activity and exercise for people living with CKD is limited. Despite popularity, there is little evidence that current digital interventions yield the effects expected from traditional face-to-face interventions. However, 14 registered trials were identified which may strengthen the evidence-base.

**Supplementary Information:**

The online version contains supplementary material available at 10.1007/s10916-024-02081-z.

## Introduction

Chronic kidney disease (CKD) is a global public health challenge that affects 1 in 10 adults worldwide, incurs an annual mortality rate of 1.2 million and accounts for 35 million disability adjusted life-years [1]. CKD is a complex condition encompassing a continuum from stage 1 (mild) to 5 (kidney failure) which requires kidney replacement therapy in the form of dialysis or transplant to maintain life. CKD has a high prevalence of comorbidities such as diabetes, hypertension and heart disease [2] as well as systemic complications including mineral bone disease, neurological disorders and accelerated sarcopenia [3]. CKD is a stronger risk factor for coronary events and all-cause mortality than diabetes [4], yet it remains under-diagnosed by clinicians [5] and under-recognised by the general public with less than 10% of people with biomarkers for kidney dysfunction being aware of the condition [6].

Across the spectrum of CKD, engaging in physical activity and exercise is essential to maintain quality of life and to interrupt the cycle of deconditioning, avoid exacerbation of comorbidities and further decline in kidney function [7–9]. Importantly, exercise and physical activity can elicit improvements in health outcomes that are relevant to the severity of the disease (described in detail elsewhere [10]). Examples include improving aerobic capacity for people without kidney replacement therapy [7], enhancing physical function in haemodialysis cohorts [8] and quality of life in transplant recipients [9]. Within this review, we defined physical activity as any bodily movement produced by skeletal muscles that required energy expenditure, including structured exercise to improve health-related outcomes or incidental activities for transport or occupational means [11].

Physical activity and exercise are recognised as essential components of kidney care as evident by recent international practice recommendations [12–14]. However physical activity and exercise interventions frequently lack support in many formal health care settings [15] including lack of funding and systems to support service provision, absence of exercise practitioners in multidisciplinary care teams and limited capability to provide support within existing care teams [16, 17]. These factors highlight the need for feasible and accessible strategies to support physical activity uptake for people living with CKD.

Digital interventions have gained significant attention as a potential method to support self-management in CKD for medication [18], dietary modifications [19], physical activity [20] and general wellbeing [21]. These interventions involve information and communication technologies to streamline the delivery of healthcare services across multiple socio-geographic settings [22]. Thus, this type of intervention may also help overcome current barriers for accessing physical activity interventions for people living with CKD by providing an accessible platform for service delivery [23]. A 2023 meta-analysis found that digital physical activity interventions improved health-related quality of life, physical function and symptoms of mental illness (i.e., depression and anxiety) for people with various chronic conditions, including type 2 diabetes and cardiovascular disease [24]. However, the analysis did not include people living with CKD and to our knowledge there has been no systematic review of digital health interventions for physical activity in CKD. As such, this study aimed to systematically review the effect of digital health interventions for physical activity and exercise on health outcomes and feasibility for people living with CKD.

## Methods

This systematic review was registered with PROSPERO (registration number: CRD42022328856) and is reported in line with the Preferred Reporting Items for Systematic Reviews and Meta-Analysis (PRISMA) 2020 statement [25] (Table S1). All deviations from protocol are reported in the manuscript.

### Data Sources & Search Strategy

Eligible studies were identified through a systematic search of the electronic databases PubMed, CINAHL, Embase and Cochrane, from 1 January 2000 to 1 December 2023. The year 2000 was determined based on previous reports that digital health applications were not widely used before this time [26]. Search terms using key words, subject headings, and synonyms related to CKD, exercise and digital health were used (Table S2). Studies identified through searching relevant reference lists and conferences were also included.

### Eligibility Criteria

Studies were included if they were an original, peer reviewed research article that used a digital platform (e.g., application, phone, internet) to promote or support autonomous physical activity/ exercise for adults (aged ≥ 18 years) with a diagnosis of CKD (including pre-dialysis, kidney failure or kidney transplant). Digital interventions included technologies that transmit digital information across communication networks (e.g., applications) [27] or online platforms with on-demand physical activity/ exercise content. Studies were required to evaluate the feasibility or effect of a digital physical activity/ exercise intervention and reported health-related outcomes. Here, we defined health-related outcomes as events that occurred following a therapeutic procedure, including those observed by a healthcare professional or self-reported by participants [28]. Interventions which included exercise programming or education as part of a broader behavioural intervention (e.g., diet modification, counselling) were eligible, however intervention components were not separated. There was no restriction on language, comparator group activity or co-morbidities. It should be noted that we deviated from protocol to include non-English studies (where translation was possible) and CKD of all stages rather than excluding Stages 1–2 because of the grouping of cohorts (e.g., Stage 1–4) as well as the low number of studies available.

We excluded review articles (systematic or literature), articles that did not evaluate a digital intervention, studies where only synchronous supervision (e.g. in-person or videoconferencing) was used to deliver the intervention [29], digital interventions that did not promote physical activity or exercise, or studies where no full text was available.

### Study Selection

After removal of duplicates, the titles and abstracts of potentially eligible studies were independently screened in duplicate (MEL, TBT, SF or RA). Studies that did not meet eligibility criteria were excluded. The full texts of the remaining studies were retrieved and independently screened in duplicate (MEL, TBT, SF or RA) according to eligibility criteria. All final eligible studies were included in the review. Any discrepancies were resolved through discussion (MEL, TBT, SF, MAW or RA) until a consensus was reached. Covidence (Covidence, Melbourne, Australia), a web-based collaboration platform, was used to streamline the study selection process [30].

### Data Extraction

Two reviewers (MEL and SF or TBT) independently extracted data relating to participant and study characteristics (including study design and sample size), exercise programming, digital platform type, reported health outcomes and results. Disagreements were resolved through discussion with a third independent reviewer (TBT, MAW or RA). No missing data were encountered. No data transformations were necessary. Data for all health-related outcomes were gathered (e.g., physical fitness, step count, gait speed, mental health, quality of life), including all for time frames and analyses.

### Risk of Bias Assessment

Study quality and risk of bias were independently assessed by two reviewers (MEL, SF or TBT). Disagreements were resolved through discussion with a third reviewer (MAW or RA). Randomised control trials (RCT’s) were assessed using the Cochrane risk of bias 2 (RoB 2) tool which comprises of 5 domains [31]. These domains assess randomisation, deviations from intended interventions, missing data, outcome measurements and selective reporting, with an overall judgement for each study as ‘High,’ Low’ or ‘Some concerns’ [31].

For single-arm studies, the NIH Quality Assessment Tool for Before-After (Pre-Post) Studies with No Control Group was used [32]. This tool consists of 12 criteria which are each rated as ‘Yes’, ‘No’, or ‘Other – cannot determine, not reported, not applicable’. The overall quality of each study is then reported as ‘Good’, ‘Fair’, or ‘Poor’. Studies were not excluded based on quality appraisal.

### Data Synthesis

Due to the heterogeneity of intervention types and outcome data, a quantitative analysis was not appropriate and thus a narrative synthesis was conducted [33]. Studies were grouped according to study design (i.e. RCT, non-RCT) and digital intervention type (e.g. smartphone or associated software/ application), wearable device, computer/ website, or multiple components (Table S3 [18, 34]). The technology functionality framework was used to describe the functions of each type of technology according to seven main functions: inform, instruct, record, display, guide, remind/ alert or communicate (Table S4 [35]). Data were synthesised according to reported outcome measures using mean difference between intervention versus control group (for RCT’s), or pre versus post scores for single-arm studies. The minimal clinically important difference (MCID) was reported where data was available.

## Results

**Fig. 1:**
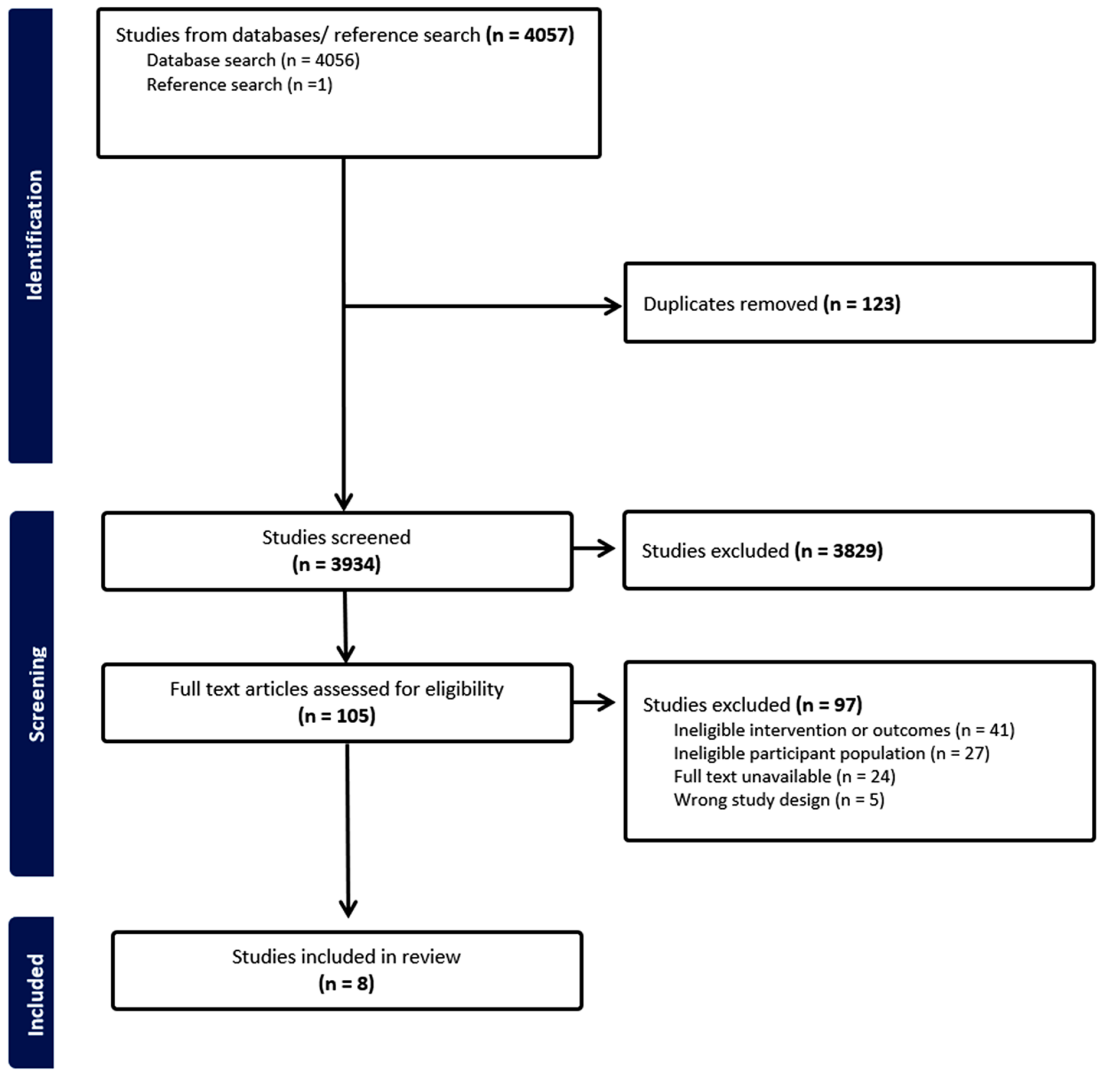
Preferred Reporting Items for Systematic reviews and Meta-Analyses (PRISMA) flow diagram of search process and study selection

The search identified 4,057 records. Following duplicate removal, the title and abstract of 3,934 articles were screened and the full texts of 105 articles were assessed for eligibility. Finally, eight studies (four RCT’s [36–39]) and four single arm [40–43]) with baseline sample sizes ranging from *n* = 17 [38] to *n* = 340 participants [39], resulting in 550 participants were included in the review (Fig. 1). Fourteen ongoing trials were also noted [44–52].

### Study Characteristics

The included studies originated from a range of regions, including North America (United States *n* = 1), Western Europe (United Kingdom *n* = 3, Ireland *n* = 1, Switzerland *n* = 1) and East Asia (Taiwan *n* = 1, South Korea *n* = 1) (Table 1). All participants were community-dwelling and aged from 18 to 90 years old. Five studies reported comorbidities, with diabetes and cardiovascular disease commonly seen in these cohorts [37–41]. The stages of CKD ranged from 1 through to haemodialysis and kidney transplant recipients (Table 1). Reported primary outcomes included self-management of CKD reported via self-report questionnaires [37, 41] physical and psychological outcomes [36, 39] and feasibility and acceptability [38, 40, 42, 43] (Table 1).

**Table 1:** Participant and study characteristics including health outcomes of each study included in this review

Author, year, country	CKD stage	Baseline mean age (years)	Sample size (sex %) at baseline and follow-up	Outcomes (observed measures)	Outcomes (self-reported measures)
*Randomised control trials*					
Castle et al., 2022, [38] United Kingdom	Single kidney transplant recipient < 3 months	Int: 39.0 (33.0–44.0) ^#^ Cont: 59.5 (53.5–65.0) ^#^	0 wk: 17 (41% F) 12 wk: 15 52 wk: 13	NR	NR
Greenwood et al., 2023, [39] United Kingdom	2–5 (including dialysis & transplant)	Int: 53.9 (13.6) Cont: 53.8 (13.5)	0 wk: 340 (46% F) 12 wk: 268	↑ 60-sec sit-to-stand: 3.4 vs. -0.4, *P* < 0.0001 ↔ Body mass (kg): 0.2 vs. 0.1, *P* = 0.61 ↔ Haemoglobin: 0.8 vs. 0.1, *P* = 0.30 ↔ Estimated glomerular filtration rate: -0.4 vs. 0.5, *P* = 0.72	↑ KDQoL-SF Mental: 2.4 vs. -1.1, *P* < 0.0001 † ↔ KDQoL-SF Physical: 1.1 vs. 1.6, *P* = 0.35 ↔ EQ-5D-5 L: 0.02 vs. -0.01, *P* = 0.64 ↔ Chalder Fatigue Scale: -1.8 vs. -1, *P* = 0.33 ↑ PAM-13: 4.3 vs. – 3.2, *P* < 0.0001 † ↔ GPAQ (MET min/week; PA min/day): -267 vs. -580, *P* = 0.29; -9.5 vs. -20.7, *P* = 0.29 ↔ PHQ-4: 0.5 vs. 0, *P* = 0.082 ↔ Work and Social Adjustment Score: -0.9 vs. -0.9, *P* = 0.49
Ki et al., 2020, [36] Korea	Haemodialysis	Range: 18 - >70 Cannot determine mean age.	0 wk: 66 12 wk: 63 (41% F)	↑ Left grip strength (kg): 4.22 vs. 0.43, *P* = 0.03 † ↑ Right grip strength (kg): 2.88 vs. -1.76, *P* = 0.048 ↑ 2-min step test: 25.45 vs. 4, *P* < 0.001 ↑ 30-sec sit to stand: 3.88 vs. 0.34 *P* < 0.001 ↔ BP (mmHg): -3.12 vs. -0.33, *P* = 0.702 ↔ Dialysis adequacy: -0.04 vs. -0.05, *P* = 0.838	↑ IPAQ (MET min/week): • Vigorous: 286.06 vs. -80, *P* = 0.007 • Moderate: 511.51 vs. -16, *P* < 0.001 • Total: 1919.57 vs. 3, *P* < 0.001 ↔ IPAQ (MET min/week): • Walking: 822 vs. 99, *P* = 0.059 ↑ Self-efficacy (exercise; outcome expectations): 10.24 vs. 3.95, *P* = 0.01; 0.31 vs. 0.04, *P* = 0.006 ↔ QoL (physical; mental): 0.59 vs. 1.54, *P* = 0.512; 3.08 vs. 1.07, *P* = 0.264
Li et al., 2020, [37] Taiwan	1–4	Int: 50.6 (11.9) Cont: 51.9 (10.2)	0 wk: 60 12 wk: 49 (27% F)	↔ Daily step count (*intervention group pre vs. post only)*: 9768.56 vs. 11389.12, *P* = 0.10 ↔ BMI (kg/m2): 0.12 vs. 0.68, *P* = 0.67 ↔ Body weight (kg): 0.41 vs. 0.09, *P* = 0.59 ↔ Body fat (%): -0.46 vs. 0.18, *P* = 0.33 ↔ Basal metabolic rate: 0.48 vs. 28.04, *P* = 0.57	↑ QoL (physical; all subscales): 1.36 vs. -0.13, *P* = 0.02; 4.24 vs. -8.67, *P* = 0.02 ↑ Self-efficacy (exercise; lifestyle): 0.2 vs. 1.04, *P* = 0.02; 0.44 vs. -1.45, *P* = 0.005 ↑ Self-management: 1.28 vs. -1.62, *P* = 0.004
*Single-arm studies*					
Anand et al., 2021, [40] United States	3b-4	58.1 (9.9)	0 wk: 28 (53.6% F) 8 wk: 19 16 wk: 16	↔ Daily step count (steps/day): • 8 wks: − 730.95 • 16 wks: − 946.1 ↔ Daily distance (m): • 8 wks: -492.6 • 16 wks: -635.7 ↔ 6MWT (m): • 8 wks: 20.6 • 16 wks: 12.2 ↔ Handgrip strength (kg): • 8 wks: 0.2 • 16 wks: 0.3 ↔ Waist circumference (cm): • 8 wks: 2.9 • 16 wks: 1.6 ↔ Body weight (kg): • 8 wks: -0.9 • 16 wks: -0.1 ↔ Blood pressure (mmHg): • 8 wks: 1 • 16 wks: 4	↑ MVPA (mins/day): • 8 wks: -2.3 • 16 wks: -2.2 ↔ Light PA (mins/day): • 8 wks: -8.5 • 16 wks: -9.7 ↔ Mental health (depression; resilience): • 8 wks: 0.9; -0.1 • 16 wks: 0.8; -0.1 ↔ Exercise self-efficacy (sticking to it; making time): • 8 wks: -0.4; -0.1 • 16 wks: -0.5; -0.3 ↔ QoL (physical; mental): • 8 wks: -1.9; -1.3 • 16 wks: -1.8; -1.5
Doyle et al. 2019, [41] Ireland	2–5	50.1	0 wk: 23 12 wk: 20 (45% F)	↑ 6MWT (m): 512.3 vs. 542.1, Δ = 29.8, *P* = 0.022 ↓ Waist circumference (cm): 99.7 vs. 97.1, Δ= -2.6, *P* < 0.001 ↓ Body fat (kg): NR, *P* = 0.012 ↓ Total cholesterol: NR; *P* = 0.023 ↓ LDL: NR; *P* = 0.005	↔ IPAQ (MET min/week) 2474.5 vs. 2950.2; Δ = 475.7; *P* > 0.05
Mayes et al., 2021, [42] United Kingdom	All stages	NR	0 wk: 276 26 wk: 85 (NR)	NR	Achieving 150 min/week of moderate intensity physical activity; 100% increase^*^ Achieving 75 min/week of vigorous-intensity physical activity; 20% increase^*^ Achieving twice-weekly strength training; 74% increase^*^ Perceived energy levels to be good or very good; 50% increase^*^
Zemp et al., 2022, [43] Switzerland	Haemodialysis	77.2	0 wk: 21 (38% F) 12 wk: 14	NR	NR

↑ statistically significant increase; ↓ statistically significant decrease; ↔ no statistically significant change; † minimal clinically important difference. Data reported is difference within intervention vs. control group for RCT’s or pre vs. post for non-RCT’s and p-value, unless otherwise stated. ^#^ Data for Castle et al. are reported as median (interquartile range) for continuous data or number (percentage) for categorical data for intervention vs. control group. ^*^self-report with no statistical analysis only percentage change reported. Studies are listed in order of study type (RCT’s first, then single arm) and intervention components. Note: data reported for Anand et al. and Greenwood et al. is intention-to-treat analysis, although as-treated and per-protocol analysis was also performed and this has been reported in-text. Units of measurement for all time-based tests (e.g. 30-sec sit-to-stand) is number of repetitions

*Abbreviations 6MWT* 6-minute walk test [88]; *BMI* body mass index; *BP* blood pressure; *DEMMI* de Morton Mobility Index [89]; *EQ-5D-5 L* Euro-Quality of Life 5 dimensions 5 levels questionnaire [90]; *F* female; *GPAQ* Global Physical Activity Questionnaire [55]; *IPAQ* International Physical Activity Questionnaire [53]; *KDQoL* kidney disease quality of life instrument [88]; *KDQoL-SF* Short form of the kidney disease quality of life instrument [88]; *LDL* low-density lipoprotein; *MET* metabolic equivalent of task; *M* male; *min* minute; *NR* not reported; *OEE* outcome expectations for exercise [60]; *PA* physical activity; *PAM* patient-activation measure [73]; *PHQ-4* Patient Health Questionnaire-4 [91]; *QoL* quality of life; *RCT* randomised control trial; *sec* seconds; *SEE* self-efficacy for exercise [92]; *SPPB* Short Physical Performance Battery [88];*T1* baseline timepoint; *T2* follow-up timepoint; *TUG* timed up and go [88]; *wks* weeks

It should be noted that Anand et al. was treated as a single-arm study as only the control group met inclusion criteria of this review with pre post data presented. Further, the RCT by Castle et al. [38] and single arm study by Zemp et al. [43], with primary outcomes of feasibility, reported health-related outcomes without undertaking significance testing of these outcomes. As such, we have reported the health-related outcomes, but these were not able to contribute to the evidence for the effectiveness of digital interventions on health outcomes. In addition, preliminary pilot data reporting on usability and acceptability of the Kidney Beam website was reported by Mayes et al. [42] and subsequently investigated in an RCT by Greenwood et al. [39]. Both studies were included in this review.

### Intervention Summary and Co-Design

The average intervention duration was 22 weeks, ranging from 12 weeks [36, 41] to one year [38]. Digital intervention type varied, with two studies investigating multiple component types (wearable device and smartphone [37, 40] three studies evaluating smartphone applications (one RCT and two single-arm) [36, 41, 43] and three studies evaluating a website (two RCT and one single-arm study) [38, 39, 42] (Table 2). Of the two studies using wearable devices, both had smart wristbands that measured step count [37, 40] while Li et al. also measured calories and sleep. However, neither reported wear time protocol. Most were stand-alone digital interventions, although Anand et al. [40] compared a lone digital intervention against a digital intervention combined with face-to-face training by fitness professionals. Similarly, Zemp et al., [43] included an initial face-to-face session followed by a digital program (Table 2).

**Table 2:** Description of the interventions, including intervention type, features, and functionality

Study	Duration (weeks), type	Intervention description	Digital technology function
*Multiple component (wearable device + smartphone application)*			
Anand et al. [40]	16, PA + EX	The intervention group received a 30-minute face-to-face behaviour change counselling session (including goal setting), an activity tracker, app to record step count, encouragement to achieve 100–150 min p/w of moderate physical activity, twice weekly 1-hour face-to-face group exercise sessions and weekly phone calls or text messages. The control group received the same counselling session, activity tracker, app, encouragement and weekly phone/ text interactions. The control group did not receive group exercise sessions.	Record; Display; Communicate
Li et al. [37]	12, PA	The intervention group received an activity tracker (to collect exercise data) and app (to record diet diary). Diet, exercise and self-management education were provided. Daily text messages, a social media support group and study-set step goals were provided. The control group received an activity tracker and app with routine care. They did not receive education or support group access.	Inform; Display Record; Communicate
*Smartphone application*			
Ki et al. [36]	12, EX	The intervention group received an app-delivered exercise program consisting of flexibility (5 min, 3x p/w), aerobic (10 min, 3x p/w increasing to 30 min) & resistance (10–15 min, 3x p/w and increasing to 20 min) training. Participants reported intensity, which gradually increased. The app included videos, education, goal setting and support. Weekly text messages and a one-off individual phone call were provided. The control group received education and exercised independently.	Inform; Instruct; Record; Display; Remind/Alert; Communicate
Doyle et al. [41]	12, PA	All participants used the ‘MiKidney’ app, which stored medical details, provided education (on CKD, medication, diet, healthy lifestyle), tracked daily exercise, set weekly goals, delivered reminders, feedback and motivational messages.	Inform; Record; Display; Remind/Alert
Zemp et al. [43]	12, EX	All participants received an initial face-to-face session with a physiotherapy examination and access to the ‘Fit’ app that contained a bank of 34 resistance, balance and mobility exercises of varying difficulties. The physiotherapist tailored the program by selecting 4–6 exercises, monitored participant exercise data and provided instructions. Participants self-reported exercise difficulty. Weekly remote communication and automatically generated feedback was provided.	Instruct, Record, Display, Guide, Communicate
*Computer system (websites)*			
Castle et al. [38]	52, PA + EX	The intervention group independently completed 12 weekly sessions using the ‘ExeRTiOn’ (exercise in renal transplant online) website, which included interactive activities, education, exercise diary, self-reported physical activity and body weight, individualised goal setting and two-way message functions. Personalised reminders were sent if 2 consecutive sessions were missed. The control group received routine care, dietary advice, and encouragement to engage in physical activity and healthy eating.	Inform, Record, Display, Remind/Alert, Communicate
Mayes et al. [42]	26, EX	All participants used the ‘Kidney Beam’ website which consisted of live and recorded exercise training and educational videos. Emails and blogs were used for feedback and encouragement.	Inform; Instruct; Communicate
Greenwood et al. [39]	12, EX	The intervention group used the ‘Kidney Beam’ website which included live and pre-recorded exercises. Each session included mobility and stretching exercises (2 × 10 min), moderate intensity aerobic and resistance training (20–30 min) and CKD specific education (15 min). Physiotherapists encouraged participants to achieve 150 min of moderate or 75 min of vigorous aerobic activity and twice weekly resistance training. The waitlist-controlled group received usual care.	Inform, Instruct, Communicate

*Abbreviations EX* exercise; *min* minute; *PA* physical activity; *p/w* per week

Digital interventions varied from the use of commercially available to custom-made applications, while four studies [36, 38, 39, 41] utilised a co-design process. Doyle et al. [41] conducted focus groups with people living with CKD (*n* = 8) to obtain feedback and inform the development of their application. Ki et al. [36] included consultation processes, including patient surveys (*n* = 40), multidisciplinary kidney care team input and consultation with application developers. The *ExeRTiOn* website from Castle et al. [38] was refined following an initial qualitative study which provided usability feedback from kidney transplant recipients (*n* = 11) and care providers (*n* = 6). Similarly, the *Kidney Beam* website in Greenwood et al. [39] was refined using feedback from people living with CKD in their preliminary study as reported by Mayes et al. [42]. Studies by Li et al. [37], and Zemp et al. [43] used pre-existing applications the WowGoHealth (GSH AI health platform) [37] and Fit application (Dividat AG, Schindellegi, Switzerland) [43] respectively while Anand et al. [40] employed a custom-made application but did not incorporate co-design.

### Risk of Bias Assessment

The risk of bias of the four RCT’s [36–39] was assessed using the Cochrane RoB2 tool [31]. Overall, all four were considered at high risk of bias. Risk of bias in the randomisation process domain was high for Castle et al. [38] and Ki et al. [36], while Li et al. [37] had some concerns and Greenwood et al. [39] had low risk for this domain. Both Castle et al. [38] and Greenwood et al. [39] had low risk of bias for deviations from intended intervention while Li et al. [37] was considered high risk and Ki et al. [36] had some concerns. All four RCT’s were considered low risk of bias for missing outcome data, high risk for the outcome measurement domain. Greenwood et al. [39] was the only RCT with a low risk of bias in the selective reporting domain, with the other three studies showing some concerns (Figure S1).

Risk of bias of the four single-arm studies [40–43] was assessed using the NIH quality assessment tool [32]. Generally, there was a lack of clarity regarding the reporting outcome measures at multiple time points [40–43]. Anand et al. [40], Zemp et al. [43] and Doyle et al. [41] clearly reported study objectives and intervention details. Anand et al. [40] and Zemp et al. [43] clearly reported eligibility criteria. Zemp et al. [43] was the only study to report blinding of outcome assessors while Doyle et al. [41] was the only study to include a representative population. Anand et al. [40] and Doyle et al. [41] both reported low attrition and appropriate statistical analysis. The remaining risk of bias domains were either not applicable, could not be determined or were not reported (Table S5). Overall study quality was considered fair for Anand et al. [40], Doyle et al. [41] and Zemp et al. [43], and poor for Mayes et al. [42]. This could be due to Mayes et al., reporting on preliminary data from a rapid rollout of an intervention in a letter to the editor format [42]. This information was used to guide the interpretation of risk of bias findings.

### Physical Activity Outcomes

All studies assessed changes in physical activity level, though reporting tools differed. The International Physical Activity Questionnaire (IPAQ) [53] was reported in two studies [36, 40] and one single arm study [41]. Ki et al. [36] reported significant improvements for total (mean difference 1619.57 vs. 3.00, *P* < 0.001), vigorous (286.06 vs. 48.00, *P* = 0.007) and moderate (511.51 vs. -16.00, *P* < 0.001) metabolic equivalent of task (MET) minutes/week [54] for intervention versus control group respectively. Anand et al. [40] and Doyle et al. [41] did not show any significant changes in the IPAQ. Daily step count and distance travelled were reported in Anand et al. and Li et al., but neither reported significant changes (Table 1) [37, 40]. Similarly, Greenwood et al. [39] found no significant changes in Global Physical Activity Questionnaire [55]. Castle et al. [38] reported on the General Practice physical activity questionnaire [56] without significance testing.

Intensity of physical activity was reported in one study by Anand et al. [40] Minutes of moderate-vigorous physical activity per day showed no significant change after eight and 16 weeks (mean difference − 2.3 and − 2.2 respectively). Minutes of daily light physical activity improved at eight weeks in as-treated analysis only. There were no changes for either analysis at 16 weeks [40] (Table 1).

Mayes et al. [42] included participant self-reported physical activity levels and whether they were meeting the physical activity guidelines. This study found a greater number of people achieving 150 min per week of moderate intensity activity (100% increase) and 75 min/week of vigorous intensity (20% increase) physical activity, as well as a 74% increase in those achieving twice-weekly strength training sessions [42].

### Physical Function Outcomes

A range of physical function outcomes were measured (Table 1). Handgrip strength was reported in two studies [36, 40] with only Ki et al. showing statistically significant improvements for left (2.88 vs. -1.76, *P* = 0.048) and right grip-strength (4.22 vs. 0.43, *P* = 0.030) in the intervention group compared with control [36]. Left grip strength reached the threshold for MCID for people undergoing haemodialysis (i.e., 4.24 kg) [57] (Table 1). The 6-minute walk test (6MWT) was reported in two studies [40, 41] with only Doyle et al. showing improvements (*P* = 0.022) [41]. Variations of the sit-to-stand test, including 60-seconds and 30-seconds, were reported by Greenwood et al. [39] and Ki et al. [36], respectively. Both studies demonstrated improvements (Greenwood et al. 3.4 vs. -0.4, *P* < 0.0001) (Ki et al. 3.88 vs. 0.34, *P* < 0.001). Ki et al. also showed improvements in the two-minute step test (25.45 vs. 4.00, *P* < 0.001) [36] (Table 1).

Two studies reported on physical function but did not perform significance testing, including the 6MWT by Castle et al. [38] and the 4-metre walk, handgrip strength, Short Physical Performance Battery, timed up and go, 60-seconds sit-to-stand and de Morton Mobility Index by Zemp et al. [43].

### Body Composition & Clinical Outcomes

A range of anthropometric variables were reported with mixed findings. Body weight showed no significant change in Anand et al., Greenwood et al. and Li et al. [37, 39, 40]. Waist circumference was significantly reduced in Doyle et al. (pre: 99.7 vs. post: 97.1, *n* = 20, *P* < 0.001) [41] although Anand et al. reported no significant changes in this outcome. Body fat significantly decreased in Doyle et al. (pre vs. post not reported, *n* = 20, *P* = 0.012) [41] however Li et al. reported no significant changes in this outcome (Table 1). Castle et al. reported waist and hip circumference, body weight, body mass index, fat and lean muscle mass outcomes without significance testing.

Clinical outcomes were not commonly reported. Doyle et al. demonstrated significant improvements in total and low-density lipoprotein cholesterol levels [41]. Anand et al. and Ki et al. showed no differences in blood pressure control [36, 40] and dialysis adequacy, respectively [36]. Similarly, Castle et al. reported blood pressure, arterial stiffness and resting heart rate measures without significance testing [38] (Table 1).

### Self-efficacy, Quality of Life and Patient-Activation

Self-efficacy was reported in three studies with a range of instruments used. Ki et al., used the Self-efficacy for Exercise scale and found significant improvements for intervention groups compared to control (10.24 vs. 3.95, *P* = 0.01) [36]. Li et al. used an unspecified scale and also found significant improvements in self-efficacy for exercise (0.2 vs. 1.04, *P* = 0.02) and lifestyle subscales (0.44 vs. -1.45, *P* = 0.005) [37]. Anand et al. reported no significant change in exercise self-efficacy [40].

Findings on quality of life (QoL) were mixed. The Kidney Disease Quality of Life (KD-QoL) Instrument was reported in three studies. Li et al. found significant improvements in the physical function subscale for intervention versus control (1.36 vs. -0.13, *P* = 0.02) [37]. Greenwood et al. showed significant improvements in the mental subscale for the intervention group (2.4 vs. -1.1, *P* < 0.0001) while Ki et al. showed no significant changes. Greenwood et al. also assessed QoL using the EuroQol- 5 dimensions- 5 levels questionnaire (EQ-5D-5L) which demonstrated no significant changes (0.02 vs. -0.01, *P* = 0.64) [39] (Table 1). Anand et al. reported physical and mental subscales of the 12-Item Short Form Health Survey (SF-12) with no significant changes.

Li et al. used a self-management questionnaire developed by the authors that showed significant improvements for intervention compared to control [37] (1.28 vs. -1.62, *P* = 0.004). Greenwood et al. used the Patient Activation Measure 13-item (PAM-13) [58] and showed significant improvement for intervention compared to waitlist-control (4.3 vs. – 3.2, *P* < 0.0001) [39]. This difference also exceeded the threshold for MCID (≥ 4-point difference) [59]. Perceptions of health were reported in Mayes et al., with improvement in perceived energy levels to be either good or very good [42].

Outcome efficacy expectations, a measure of the beliefs of older adults regarding the benefits of exercise, were assessed by Ki et al. [36]. Improvements were reported using the Exercise Outcomes Expectations questionnaire [60] (0.31 vs. 0.04, *P* = 0.006) for the intervention group compared to control. Fatigue, mental well-being and social functioning were assessed in Greenwood et al. [39] with no changes reported.

Fatigue, self-efficacy for exercise and nutrition, and health related QoL were included in Castle et al. [38] and physical and mental QoL reported in Zemp et al. [43]. However, no significance testing was conducted on these outcomes due to the primary aim of Castle et al. and Zemp et al. focusing on feasibility.

### Usability, Acceptability & Feasibility

Four studies reported usability and acceptability outcomes [37, 41–43]. In Li et al., 76% of participants (*n* = 19/25) gave positive feedback about the intervention, while only 24% (*n* = 6/25) found that the wearable activity tracker was inconvenient, though the method of collecting this data was not reported [37]. Participants in Doyle et al. reported that the medication list, reminders, diet and exercise information, and exercise tracker capabilities were the most beneficial features of the application [41]. The acceptability of the Fit application used by Zemp et al. [43] was evaluated using the Technology Acceptance Model questionnaire. Participants rated the acceptability of the Fit application as high to very high across four categories including perceived ease of use (mean ± standard deviation, maximum of 7 points; 6.2 ± 0.3), perceived usefulness (5.5 ± 0.4), positive attitude towards using (5.6 ± 0.4) and behavioural intention to use (3.9 ± 0.6) [43]. Finally, Mayes et al. found that 96% of participants (*n* = 82/85) would recommend their website [42].

Feasibility outcomes were reported in three studies [38, 40, 43]. Castle et al. achieved all *a-priori* criteria for feasibility, including screening rate of 84.2% (95% CI: 68.6–94.0; target: >50%), recruitment rate of 62.5% (95% CI: 43.7–79.0; target: >50%), retention at study completion of 76.4% (95% CI: 50.0-93.2; target: >60%) and intervention adherence of 66% (95% CI 29.9–92.5; target: >60% of sessions completed) [38]. Six unrelated adverse events were reported throughout the 12-month study duration, and these occurred evenly across the intervention and control groups (3 participants in each group). Positive features of Castle et al.’s *ExeRTiOn* website included access to advice and social support, and the self-monitoring and gradual increase of physical activity. Anand et al. reported adherence and safety data as indicators of feasibility [40]. This study found high adherence to wearable activity trackers, with > 86% of participants logging activity data. Zemp et al. reported rates of inclusion (*n* = 86/197, 44%), recruitment (*n* = 22/86, 26%), attrition (*n* = 6/21, 29%) and exercise adherence (*n* = 17/24, 73%) [43]. Participants’ exercise adherence was lower than the acceptability threshold of ≥ 75% which was attributed to health related reasons (*n* = 4), difficulties using tablet computer (*n* = 2), preference for outdoor exercise, time constraints, loss of motivation (*n* = 1 each, respectively). Six unrelated adverse events were reported.

## Discussion

This study systematically reviewed the current literature on digital interventions that promote physical activity and exercise for people living with CKD. The resultant studies were few and heterogeneous in nature with varying intervention types, CKD stages and outcomes measured. The findings demonstrated insufficient evidence for physical activity levels, self-efficacy, body composition, physical function, and psychological outcomes to draw conclusions regarding the effects of digital interventions on these domains. Nonetheless, these studies demonstrated feasibility and acceptability for digital interventions in this cohort, which may be related to the use of co-design. The limitations of the findings from this review may be attributed to the small number of included studies (*n* = 8) and typically high risk of bias. Although the current evidence base is small, fourteen registered clinical trials were identified which may strengthen the current evidence-base in the near future, notably the SMILE-K [61] and SUCCESS [62] trials which are nearing completion with more than 300 participants each.

This systematic review addresses the gap in knowledge regarding digital physical activity interventions for people with CKD. Recent systematic and Cochrane reviews suggest the potential efficacy for digital interventions to improve other components of self-management including dietary modification (e.g., sodium and fluid intake), medication adherence and symptom management [18, 19, 63]. The results of this review suggest that digital interventions promoting physical activity for CKD were feasible and acceptable. This is in line with previous systematic reviews of digital health interventions to promote physical activity/ exercise for cohorts such as people with complex health needs including adult cancer survivors [64, 65] and older adults [66].

The utilisation of a co-design process through collaboration with people living with CKD and kidney care teams was observed in 50% of the studies included in this review [36, 38, 39, 41] which may have contributed to high levels of acceptability. However, improvements in health-related outcomes such as aerobic capacity, muscular strength and wellbeing as seen with face-to-face exercise interventions [7–9] were not apparent. Heterogeneity of outcome measures made synthesising data and comparing studies challenging. Diversity of outcome measures is a common trait among exercise trials for people living with CKD and was also highlighted in a 2021 systematic review by Jegatheesan et al. [67]. Similar to Jegatheesan et al. [67], we found the most commonly used outcomes were the physical function component of QoL questionnaires, hand grip strength and the 6MWT, none of which showed consistent change across studies. The use of standardised outcome measures that address clinical priorities such as fall risk and poor mobility [68] would facilitate data pooling and enable accurate comparisons between studies [69]. Future studies may consider outcomes such as the Short Physical Performance Battery [70] and the mobility component of the Integrated Palliative Outcome Scale-Renal [71] to assist in quantifying effect sizes and determining clinical importance.

The use of behaviour change models was a strength of several studies in this review. In particular, the Behaviour Change Wheel [39, 42], the Theory of Self-Efficacy [36] and Exercise is Medicine framework [40] were used. The Behaviour Change Wheel showed improved patient-activation and achievement of physical activity guidelines across the spectrum of CKD, while the Theory of Self-Efficacy improved a range of health outcomes for people receiving maintenance dialysis. As such, clinicians working to overcome motivational challenges may benefit from using Behaviour Change Wheel or the Theory of Self-Efficacy.

Four studies reported their exercise program in detail [36, 39, 40, 43]. These programs generally consisted of aerobic, resistance, flexibility and balance training. Of note, the exercise programs by Ki et al. [36] and Zemp et al. [43] were personalised, with both studies using participant reported exertion to guide exercise progression and Zemp et al. [43] also individually tailoring exercises to each participant. In addition, Greenwood et al. [39] provided on-demand access to exercises which enabled an individualised approach. Individualisation is an important determinant of exercise adherence in people with chronic diseases [72] and may have contributed to the significant improvements in physical function outcomes in these studies. A 2023 meta-analysis attributed successful physical activity uptake and lifestyle change for people living with CKD stages 3–5 to a combination of education, goal setting and tailored plans [73]. Furthermore, individualised notifications can increase engagement and adherence to app-based programs [74]. Another aspect that may enhance success of digital interventions is co-delivery of face-to-face interventions such as that seen in the intervention arm of Anand et al. which showed superior results to digital interventions alone [40]. Providing tailored exercise programs with individualised support may be important features for effective digital physical activity interventions.

While the findings of this review demonstrate the current evidence-base is limited, fourteen registered trials were identified, including 12 RCTs (combined target sample size ~ 3,000 participants) [45–51, 61, 62, 75–77].This suggests that the evidence base will soon increase substantially, providing further insight into efficacy, though only where consumer preferences are met [78]. People living with CKD are interested in digital interventions to manage their condition [79, 80], but frequently report a lack of specific advice [81, 82]. Well-rounded digital interventions are likely to address this important gap. A recent systematic review identified key features to support consumer needs in digital exercise interventions including supporting virtual communities of care, specific education, reminders/monitoring and crucially, the ability to individualise exercise programs [78]. There were also several studies whose primary outcomes were outside the scope of this review e.g., primary focus on diet or medication with digital support, or activity trackers only without physical activity promotion components. This highlights the disconnect in currently available digital models of care where participants may only have access to one aspect of self-management. Patients with complex health conditions such as CKD require multidisciplinary care needs and are likely to benefit from combined initiatives addressing all aspects self-management. A combined digital and person-centred approach was trialled recently by Nagel et al. [21] who incorporated cultural values (social connection, country, cultural identity) with clinical aspects (medication, symptoms) to deliver a holistic wellbeing intervention for Aboriginal and Torres Strait Islander people undergoing haemodialysis. Thus, a holistic approach may overcome disconnects in the current model of care.

Despite the potential reduction in health care costs associated with adopting digital interventions [19], no cost-analyses were reported in any of the included studies in this review. The absence of cost-analyses information is a common trait noted across digital interventions for other aspects of self-management of CKD [18]. Reporting cost information in future studies may help to quantify any financial benefits of utilising digital health interventions for promoting physical activity.

The strengths of the current review include adherence to PRISMA guidelines and a comprehensive search strategy across four main databases and relevant reference lists. However, this study is not without limitations. First, the inclusion of studies that reported on all disease stages and consequent heterogeneity of participants may limit generalisability to advanced CKD. Furthermore, it was not possible to complete a meta-analysis due to the heterogeneity of interventions and outcomes. For example, the primary outcome of Castle et al. [38] and Zemp et al. [43] was feasibility, thus these studies were not powered to detect differences for health outcomes. Neither study conducted significance testing and as such were not able to inform the effect of digital interventions on health-related outcomes. The current review did not include internet-supported telehealth interventions that involve synchronous audio-video conferencing [83] and require live supervision of end-users. There has been a rapid increase in telehealth usage arising from the Covid-19 pandemic [84]. Thus, the literature for this type of intervention may expand in coming years. Future studies reviewing the effectiveness of telehealth interventions for physical activity promotion may be beneficial.

Given the rise in patient-level costs associated with disease trajectory (estimated mean cost of $3,060 United states dollars (USD) in CKD stage 3a; $57,334USD in haemodialysis; and $75,326USD for incident transplant) [85], more studies evaluating the effects of digital interventions on health outcomes for people with advanced and complex CKD may be warranted. Similarly, the importance of supporting access to exercise and physical activity as a pillar of self-management for people living with CKD should continue to be a focus [15]. This not only represents an innovative approach for health promotion but also addresses global public health concerns and research priority areas for comprehensive kidney care and equitable care delivery [86, 87].

## Conclusion

The use of digital interventions to promote physical activity and exercise for people living with CKD is an emerging area. Currently, interventions comprising smartphone applications or combined technology (wearable device + smartphone applications) are most common. Current findings indicate minimal change in physical activity and self-efficacy, conflicting results for body composition and physical function, and no change in mental health outcomes. Promising data have been reported for feasibility and acceptability. Thus, while digital interventions present an acceptable and feasible option to overcome a service gap in physical activity and exercise in routine kidney care, the evidence so far for health-related outcomes for people living with CKD is limited. Additional high-quality studies that explicitly incorporate consumers’ needs and provide tailored programs to address clinical priorities are warranted. Registry data suggest a range of trials in the pipeline may soon address this gap.

## Electronic Supplementary Material

Below is the link to the electronic supplementary material.

Supplementary Material 1

Supplementary Material 2

## Data Availability

Data is provided within the manuscript or supplementary information files. Data extracted from included studies and all data used in analyses may be available from the corresponding author by request.
