# Supplementary material for: Digital Physical Activity and Exercise Interventions for People Living with Chronic Kidney Disease: A Systematic Review of Health Outcomes and Feasibility
Source: J Med Syst. 2024 Jul 1;48(1):63. doi: 10.1007/s10916-024-02081-z (PMC11217122; doi:10.1007/s10916-024-02081-z)
Supplement: Supplementary file 1 — Supplementary Material 1 [file 10916_2024_2081_MOESM1_ESM.docx]

# Supplementary material

**Table S1:** PRISMA 2020 Checklist for manuscript and abstract, from Page et al. 2021^20^

| **Section and Topic** | **Item #** | **Checklist item** | **Location where item is reported** |
| --- | --- | --- | --- |
| **TITLE** | | |  |
| Title | 1 | Identify the report as a systematic review. | Pg. 1 |
| **ABSTRACT** | | |  |
| Abstract | 2 | See the PRISMA 2020 for Abstracts checklist. | Abstract pg. 3 |
| **INTRODUCTION** | | |  |
| Rationale | 3 | Describe the rationale for the review in the context of existing knowledge. | 1 Introduction, pg. 4-5 |
| Objectives | 4 | Provide an explicit statement of the objective(s) or question(s) the review addresses. | 1 Introduction, pg. 5 |
| **METHODS** | | |  |
| Eligibility criteria | 5 | Specify the inclusion and exclusion criteria for the review and how studies were grouped for the syntheses. | 2.2. Eligibility Criteria |
| Information sources | 6 | Specify all databases, registers, websites, organisations, reference lists and other sources searched or consulted to identify studies. Specify the date when each source was last searched or consulted. | 2.1 Data sources & search strategy |
| Search strategy | 7 | Present the full search strategies for all databases, registers and websites, including any filters and limits used. | 2.1 Data sources & search strategy |
| Selection process | 8 | Specify the methods used to decide whether a study met the inclusion criteria of the review, including how many reviewers screened each record and each report retrieved, whether they worked independently, and if applicable, details of automation tools used in the process. | 2.3 Study Selection |
| Data collection process | 9 | Specify the methods used to collect data from reports, including how many reviewers collected data from each report, whether they worked independently, any processes for obtaining or confirming data from study investigators, and if applicable, details of automation tools used in the process. | 2.4 Data Extraction |
| Data items | 10a | List and define all outcomes for which data were sought. Specify whether all results that were compatible with each outcome domain in each study were sought (e.g. for all measures, time points, analyses), and if not, the methods used to decide which results to collect. | 2.4 Data Extraction |
|  | 10b | List and define all other variables for which data were sought (e.g. participant and intervention characteristics, funding sources). Describe any assumptions made about any missing or unclear information. | 2.4 Data Extraction |
| Study risk of bias assessment | 11 | Specify the methods used to assess risk of bias in the included studies, including details of the tool(s) used, how many reviewers assessed each study and whether they worked independently, and if applicable, details of automation tools used in the process. | 2.5 Risk of bias |
| Effect measures | 12 | Specify for each outcome the effect measure(s) (e.g. risk ratio, mean difference) used in the synthesis or presentation of results. | n/a |
| Synthesis methods | 13a | Describe the processes used to decide which studies were eligible for each synthesis (e.g. tabulating the study intervention characteristics and comparing against the planned groups for each synthesis (item #5)). | n/a |
|  | 13b | Describe any methods required to prepare the data for presentation or synthesis, such as handling of missing summary statistics, or data conversions. | n/a  No missing data |
|  | 13c | Describe any methods used to tabulate or visually display results of individual studies and syntheses. | 2.4 Data Extraction |
|  | 13d | Describe any methods used to synthesize results and provide a rationale for the choice(s). If meta-analysis was performed, describe the model(s), method(s) to identify the presence and extent of statistical heterogeneity, and software package(s) used. | n/a |
|  | 13e | Describe any methods used to explore possible causes of heterogeneity among study results (e.g. subgroup analysis, meta-regression). | n/a |
|  | 13f | Describe any sensitivity analyses conducted to assess robustness of the synthesized results. | n/a |
| Reporting bias assessment | 14 | Describe any methods used to assess risk of bias due to missing results in a synthesis (arising from reporting biases). | n/a |
| Certainty assessment | 15 | Describe any methods used to assess certainty (or confidence) in the body of evidence for an outcome. | n/a |
| **RESULTS** | | |  |
| Study selection | 16a | Describe the results of the search and selection process, from the number of records identified in the search to the number of studies included in the review, ideally using a flow diagram. | 3 Results; Figure 1 |
|  | 16b | Cite studies that might appear to meet the inclusion criteria, but which were excluded, and explain why they were excluded. | n/a |
| Study characteristics | 17 | Cite each included study and present its characteristics. | 3.1 Study characteristics; 3.2 Intervention summary; Table 1 & 2 |
| Risk of bias in studies | 18 | Present assessments of risk of bias for each included study. | 3.3 Risk of bias |
| Results of individual studies | 19 | For all outcomes, present, for each study: (a) summary statistics for each group (where appropriate) and (b) an effect estimate and its precision (e.g. confidence/credible interval), ideally using structured tables or plots. | Table 2 |
| Results of syntheses | 20a | For each synthesis, briefly summarise the characteristics and risk of bias among contributing studies. | n/a |
|  | 20b | Present results of all statistical syntheses conducted. If meta-analysis was done, present for each the summary estimate and its precision (e.g. confidence/credible interval) and measures of statistical heterogeneity. If comparing groups, describe the direction of the effect. | n/a |
|  | 20c | Present results of all investigations of possible causes of heterogeneity among study results. | n/a |
|  | 20d | Present results of all sensitivity analyses conducted to assess the robustness of the synthesized results. | n/a |
| Reporting biases | 21 | Present assessments of risk of bias due to missing results (arising from reporting biases) for each synthesis assessed. | n/a |
| Certainty of evidence | 22 | Present assessments of certainty (or confidence) in the body of evidence for each outcome assessed. | n/a |
| **DISCUSSION** | | |  |
| Discussion | 23a | Provide a general interpretation of the results in the context of other evidence. | 4 Discussion, pg. 14-17 |
|  | 23b | Discuss any limitations of the evidence included in the review. | 4 Discussion, pg. 16-17 |
|  | 23c | Discuss any limitations of the review processes used. | 4 Discussion, pg. 16-17 |
|  | 23d | Discuss implications of the results for practice, policy, and future research. | 4 Discussion pg. 15,  5 Conclusion |
| **OTHER INFORMATION** | | |  |
| Registration and protocol | 24a | Provide registration information for the review, including register name and registration number, or state that the review was not registered. | 2 Methods |
|  | 24b | Indicate where the review protocol can be accessed, or state that a protocol was not prepared. | 2 Methods |
|  | 24c | Describe and explain any amendments to information provided at registration or in the protocol. | 2.2. Eligibility criteria |
| Support | 25 | Describe sources of financial or non-financial support for the review, and the role of the funders or sponsors in the review. | Acknowledgements – pg. 19 |
| Competing interests | 26 | Declare any competing interests of review authors. | Disclosure – pg. 19 |
| Availability of data, code and other materials | 27 | Report which of the following are publicly available and where they can be found: template data collection forms; data extracted from included studies; data used for all analyses; analytic code; any other materials used in the review. | Availability of data – pg. 19 |

| **Section and Topic** | **Item #** | **Checklist item** | **Reported (Yes/No)** |
| --- | --- | --- | --- |
| **TITLE** | | |  |
| Title | 1 | Identify the report as a systematic review. | Yes |
| **BACKGROUND** | | |  |
| Objectives | 2 | Provide an explicit statement of the main objective(s) or question(s) the review addresses. | Yes |
| **METHODS** | | |  |
| Eligibility criteria | 3 | Specify the inclusion and exclusion criteria for the review. | Yes |
| Information sources | 4 | Specify the information sources (e.g. databases, registers) used to identify studies and the date when each was last searched. | Yes |
| Risk of bias | 5 | Specify the methods used to assess risk of bias in the included studies. | yes |
| Synthesis of results | 6 | Specify the methods used to present and synthesise results. | yes |
| **RESULTS** | | |  |
| Included studies | 7 | Give the total number of included studies and participants and summarise relevant characteristics of studies. | Yes |
| Synthesis of results | 8 | Present results for main outcomes, preferably indicating the number of included studies and participants for each. If meta-analysis was done, report the summary estimate and confidence/credible interval. If comparing groups, indicate the direction of the effect (i.e. which group is favoured). | Yes |
| **DISCUSSION** | | |  |
| Limitations of evidence | 9 | Provide a brief summary of the limitations of the evidence included in the review (e.g. study risk of bias, inconsistency and imprecision). | Yes |
| Interpretation | 10 | Provide a general interpretation of the results and important implications. | Yes |
| **OTHER** | | |  |
| Funding | 11 | Specify the primary source of funding for the review. | N/A |
| Registration | 12 | Provide the register name and registration number. | Yes |

**Table S2:** Search terms

|  |  |  |  |
| --- | --- | --- | --- |
| **PubMed** | **CINAHL** | **Cochrane** | **Embase** |
| 1. Renal dialysis (MESH) | 1. CKD (All Text) | 1. telemedicine (Title Abstract Keyword) | 1. ckd.tw. |
| 2. CKD (Title/Abstract) | 2. chronic kidney disease (All Text) | 2. fitness trackers (Title Abstract Keyword) | 2. hemodialysis/ |
| 3. Chronic kidney disease (Title/Abstract) | 3. kidney failure (All Text) | 3. mobile applications (Title Abstract Keyword) | 3. chronic kidney disease.tw. |
| 4. Kidney disease (Title/Abstract) | 4. renal dialysis (All Text) | 4. digital health (Title Abstract Keyword) | 4. kidney disease.tw. |
| 5. Kidney failure (Title/Abstract) | 5. kidney disease (All Text) | 5. ehealth (Title Abstract Keyword) | 5. kidney failure.tw. |
| 6. Exercise therapy (MESH) | 6. exercise (All Text) | 6. e-health (Title Abstract Keyword) | 6. 1 or 2 or 3 or 4 or 5 |
| 7 Physical activity (MESH) | 7. physical activity (All Text) | 7. mhealth (Title Abstract Keyword) | 7. exercise therapy.mp. |
| 8. Exercise (Title/Abstract) | 8. exercise therapy (All Text) | 8. m-health (Title Abstract Keyword) | 8. physical activity/ |
| 9. Behavioural intervention (Title/Abstract) | 9. behavioural intervention (All Text) | 9. mobile health (Title Abstract Keyword) | 9. exercise.tw. |
| 10. Physical health (Title/Abstract) | 10. physical health (All Text) | 10. app-based (Title Abstract Keyword) | 10 behavioural intervention.tw. |
| 11. Health outcomes (Title/Abstract) | 11. health outcomes (All Text) | 11. app (Title Abstract Keyword) | 11. physical health.tw. |
| 12. Telemedicine (MESH) | 12. telemedicine (All Text) | 12. CKD (Title Abstract Keyword) | 12. health outcomes.tw. |
| 13. Fitness trackers (MESH) | 13. Fitness trackers (All Text) | 13. chronic kidney disease (Title Abstract Keyword) | 13 7 or 8 or 9 or 10 or 11 or 12 |
| 14. Mobile applications (MESH) | 14. Mobile applications (All Text) | 14. kidney disease (Title Abstract Keyword) | 14. telemedicine/ |
| 15. Digital health (Title/Abstract) | 15. Digital health (All Text) | 15. kidney failure (Title Abstract Keyword) | 15. activity tracker/ |
| 16. Mhealth (Title/Abstract) | 16. Mhealth (All Text) | 16. renal dialysis (Title Abstract Keyword) | 16. mobile application/ |
| 17. Ehealth (Title/Abstract) | 17. Ehealth (All Text) | 17. exercise (Title Abstract Keyword) | 17. digital health.tw. |
| 18. E-health (Title/Abstract) | 18. E-health (All Text) | 18. physical activity (Title Abstract Keyword) | 18. mhealth.tw. |
| 19. M-health (Title/Abstract) | 19. M-health (All Text) | 19. exercise therapy (Title Abstract Keyword) | 19. ehealth.tw. |
| 20. App-based (Title/Abstract) | 20. App-based (All Text) | 20. behavioural intervention (Title Abstract Keyword) | 20.e-health.tw. |
| 21. Randomised control trial (All Text) | 21. App (All Text) | 21. physical health (Title Abstract Keyword) | 21. m-health.tw. |
| 22. Prospective stud* (All Text) | 22. Randomised control trial (All Text) | 22. health outcomes (Title Abstract Keyword) | 22. app-based.tw. |
| 23. Cohort stud* (All Text) | 23. Prospective stud* (All Text) | 23. 1 or 2 or 3 or 4 or 5 or 6 or 7 or 8 or 9 or 10 or 11 | 23 14 or 15 or 16 or 17 or 18 or 19 or 20 or 21 or 22 |
| 24. Feasibility stud* (All Text) | 24. Cohort stud* (All Text) | 24. 12 or 13 or 14 or 15 or 16 | 24 randomised control trial.mp. |
| 25. Pilot stud* (All Text) | 25. Feasibility stud* (All Text) | 25. 17 or 18 or 19 or 20 or 21 or 22 | 25. prospective stud*.mp. |
| 26. Clinical trial (All Text) | 26. Pilot stud* (All Text) | 26. 23 and 24 and 25 | 26 cohort stud*.mp. |
| 27. 1 or 2 or 3 or 4 or 5 | 27. Clinical trial (All Text) |  | 27 feasibility stud*.mp. |
| 28. 6 or 7 or 8 or 9 or 10 or 11 | 28. 1 or 2 or 3 or 4 or 5 |  | 28 pilot stud*.mp. |
| 29. 12 or 13 or 14 or 15 or 16 or 17 or 18 or 19 or 20 | 29. 6 or 7 or 8 or 9 or 10 or 11 |  | 29 clinical trial.mp. |
| 30. 21 or 22 or 23 or 24 or 25 or 26 | 30. 12 or 13 or 14 or 15 or 16 or 17 or 18 or 19 or 20 or 21 |  | 30 24 or 25 or 26 or 27 or 28 or 29 |
| 31. 27 and 28 and 29 and 30 | 31. 22 or 23 or 24 or 25 or 26 or 27 |  | 31 6 and 13 and 23 and 30 15 |
|  | 32. 28 and 29 and 30 and 31 |  |  |

**Table S3:** Major types of digital health interventions (Adapted from Shen et al [14] and Vegesna et al [25])

| **Type of digital health intervention** | **Description** |
| --- | --- |
| Smartphone application | Software/ application/ short message service (SMS) used to transmit patient data to physician/ researcher |
| Wearable devices | Worn or placed on a body part to record specific physiological changes (e.g., respiratory rate sensor, blood pressure monitors, heart rate) or to track physical activity data |
| Computer/ website | Computerised systems in which data are entered by the patient through the internet or are given to the patient by system |
| Multiple Component | Containing more than one technology listed above |
| Telemedicine | A planned contact that is generally prearranged between a healthcare professional and a patient for the purpose of clinical consultation, advice, and treatment planning |

**Table S4**: Technology functionality framework (From Aitken et al.[26])

| **Digital technology functions** | **Description** |
| --- | --- |
| Inform | Provide information in a variety of formats (text, photos, videos) |
| Instruct | Provide user instructions |
| Record | Collection of data entered by the user |
| Display | Graphically display of the data entered by the user/output of the data |
| Guide | Provide guidelines based on the data entered by the user |
| Remind/Alert | Provider reminders to the user |
| Communicate | Provide communication with healthcare provider/ patients and/or  provide links to social networks |

**Table S5**: Quality assessments for single-arm studies using NIH quality assessment tool for before-after (pre-post) studies with no control group

|  | **Study** | **Study question** | **Eligibility criteria and study population** | **Participants representative of clinical population** | **All eligible participants enrolled** | **Sufficient sample size** | **Intervention clearly described and delivered** | **Outcome measures clearly described, valid and reliable** | **Blinding of outcome assessors** | **Follow-up rate** | **Statistical analysis** | **Multiple outcome measures** | **Group-level and individual-level outcome efforts** | **Quality rating (Good, Fair, or Poor)** |
| --- | --- | --- | --- | --- | --- | --- | --- | --- | --- | --- | --- | --- | --- | --- |
|  | **Anand et al** | Yes | Yes | No | Other (CD) | Other (CD) | Yes | Yes | No | Yes | Yes | No | Other (NA) | Fair |
|  | **Mayes et al** | No | No | No | No | Other (CD) | No | No | No | No | No | No | Other (NA) | Poor |
|  | **Doyle et al** | Yes | No | Yes | Other (CD) | No | Yes | Other (CD) | No | Yes | Yes | No | Other (NA) | Fair |
|  | **Zemp et al** | Yes | Yes | No | Yes | Other (NA) | Yes | Other (CD) | Yes | No | No | No | Other (NA) | Fair |

Abbreviation: *CD* could not determine, *NA* not applicable


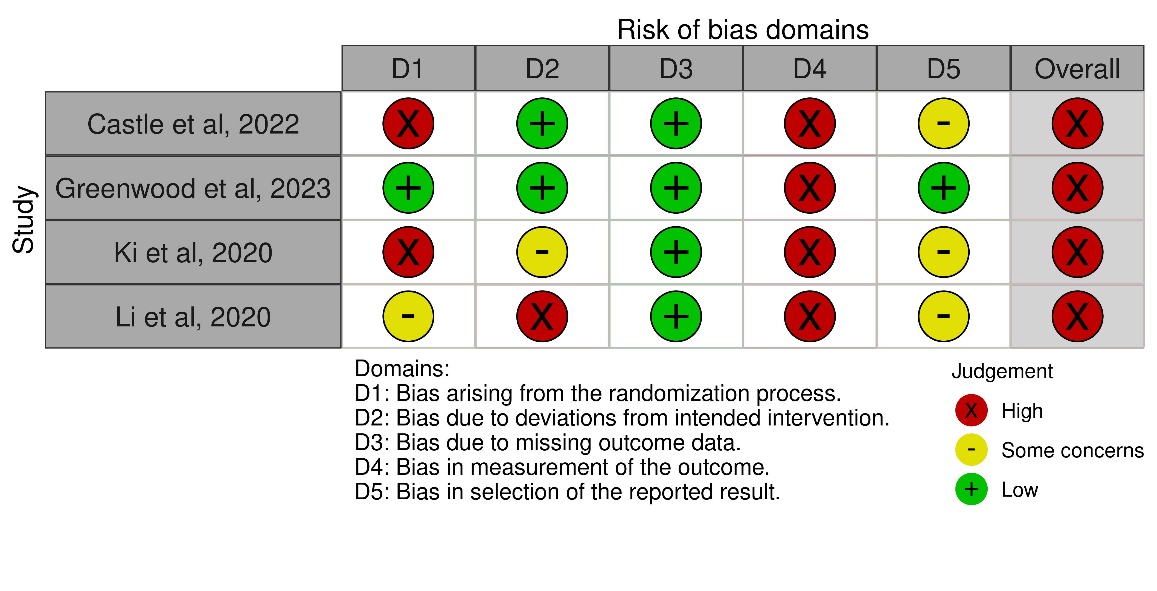
**Figure S1**: Risk of bias assessment using the Cochrane risk of bias two (RoB2) tool for assessment of randomised control trials
