# Supplementary figures and images for: Digital Physical Activity and Exercise Interventions for People Living with Chronic Kidney Disease: A Systematic Review of Health Outcomes and Feasibility
Source: J Med Syst. 2024 Jul 1;48(1):63. doi: 10.1007/s10916-024-02081-z (PMC11217122; doi:10.1007/s10916-024-02081-z)

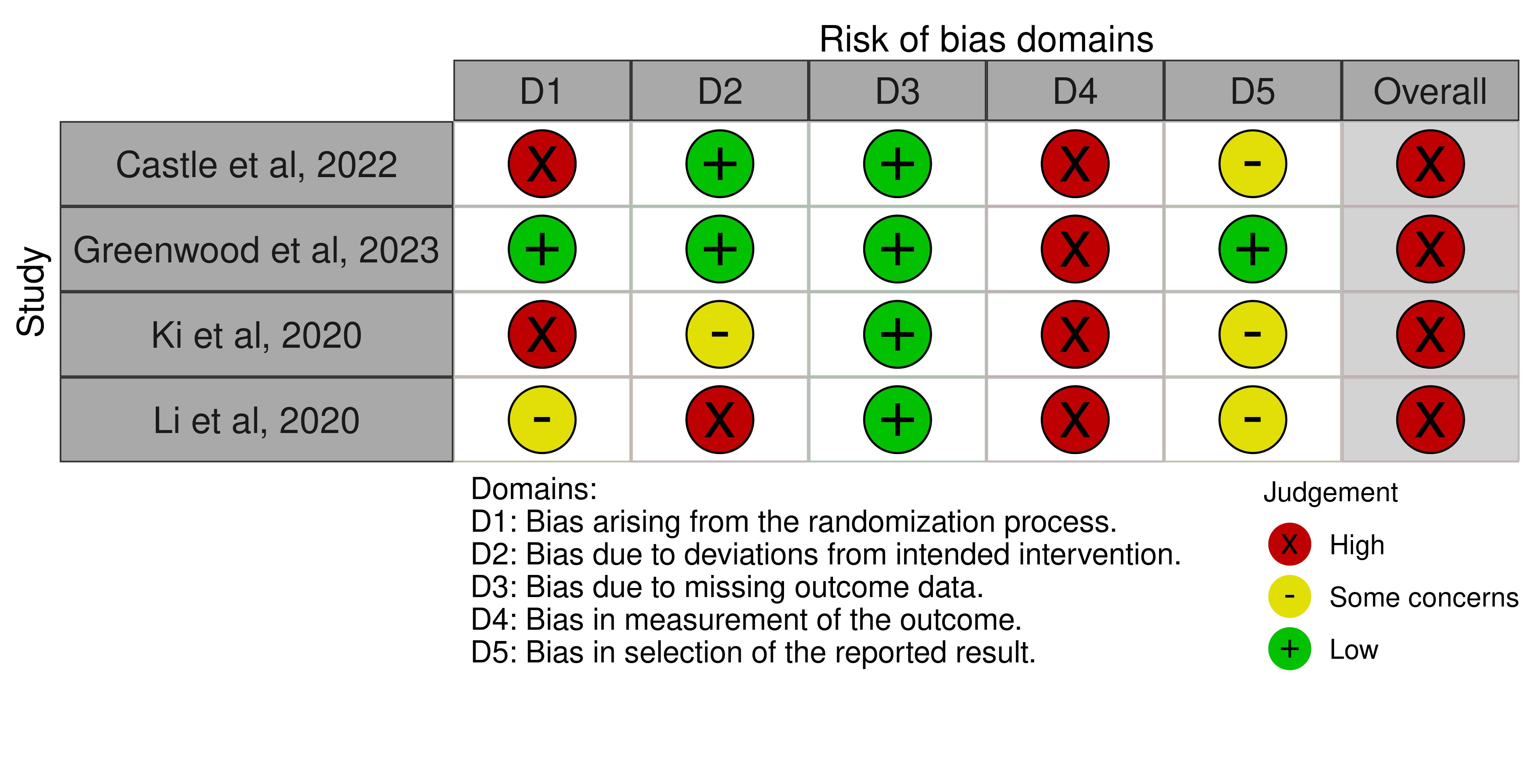

Supplement: Supplementary file 2 — Supplementary Material 2 [file 10916_2024_2081_MOESM2_ESM.jpeg]
